# Ultracompact Electrical Double Layers at TiO_2_(110) Electrified Interfaces

**DOI:** 10.1021/jacs.4c09911

**Published:** 2024-11-25

**Authors:** Immad M. Nadeem, Christopher Penschke, Ji Chen, Xavier Torrelles, Axel Wilson, Hadeel Hussain, Gregory Cabailh, Oier Bikondoa, Jameel Imran, Christopher Nicklin, Robert Lindsay, Jörg Zegenhagen, Matthew O. Blunt, Angelos Michaelides, Geoff Thornton

**Affiliations:** † London Centre for Nanotechnology and Department of Chemistry, University College London, 20 Gordon Street, London WC1H 0AJ, U.K.; ‡ Diamond Light Source Ltd., Harwell Science and Innovation Campus, Didcot, Oxfordshire OX11 0DE, U.K.; § London Centre for Nanotechnology and Department of Physics & Astronomy, University College London, 17-19 Gordon Street, London WC1H 0AH, U.K.; ∥ Institut de Ciència de Materials de Barcelona (CSIC), Campus UAB, Bellaterra 08193, Spain; ⊥ Sorbonne Université, CNRS, UMR 7588, Institut des NanoSciences de Paris, 4 Place Jussieu, Paris F-75005, France; # Department of Physics, University of Warwick, Gibbet Hill Road, Coventry CV4 7AL, U.K.; ∇ XMaS, the U.K. CRG Beamline, ESRF, The European Synchrotron, 71, Avenue des Martyrs, CS40220, Grenoble, Cedex 09 F-38043, France; ○ Corrosion and Protection Centre, Department of Materials, The University of Manchester, Sackville Street, Manchester M13 9PL, U.K.; ◆ Photon Science Institute, The University of Manchester, Manchester M13 9PL, U.K.

## Abstract

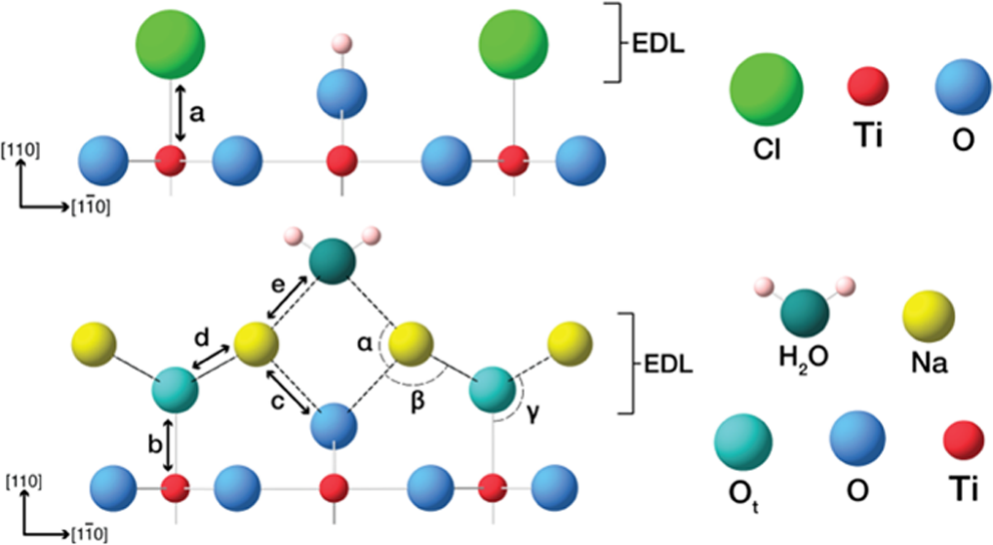

Metal-oxide aqueous interfaces are important in areas as varied as photocatalysis and mineral reforming. Crucial to the chemistry at these interfaces is the structure of the electrical double layer formed when anions or cations compensate for the charge arising from adsorbed H^+^ or OH^–^. This has proven extremely challenging to determine at the atomic level. In this work, we use a surface science approach, involving atomic level characterization, to determine the structure of pH-dependent model electrified interfaces of TiO_2_(110) with HCl and NaOH using surface X-ray diffraction (SXRD). A comparison with ab initio molecular dynamics calculations reveals the formation of surprisingly compact double layers. These involve inner-sphere bound Cl and Na ions, with respectively H^+^ and O^–^/OH^–^ in the contact layer. Their exceptionally high electric fields will play a key role in determining the chemical reactivity.

## 1 Introduction

Semiconductors are widely used in photochemistry and in photoelectro-catalysis for water splitting, with metal oxides playing a prominent role.^1−4^ The behavior of these electrified interfaces has been extensively studied, with complex mechanistic models developed to describe the multiscale processes involved.^5,6^ Electrified, or charged metal oxide interfaces are also important in colloidal and geological processes, as the pH of the electrolyte very rarely corresponds to the point of zero charge (PZC); at a pH lower than the PZC, H^+^ adsorption causes the surface to be positively charged, while at higher pH it is negatively charged due to adsorbed O^–^ or OH^–^.^5,6^

Modeling of phenomena at electrified oxide interfaces continues to require many assumptions, in particular a description of the contact layer formed between the substrate and electrolyte as well as other details of the more extended electrical double layer (EDL).^6^ Despite significant progress in addressing the gaps in our knowledge of their microscopic details, enormous challenges remain. For electrochemical interfaces of metal electrodes, it has been generally accepted that they involve intimate bonding of ions from solution in “specific adsorption”,^7^ although the detailed EDL structures are little studied. Nevertheless, there are several examples of ordered overlayers of anions electrochemically deposited onto metal single-crystal substrates.^8^ In contrast, alkaline electrolytes have been shown to exhibit nonspecific adsorption on metal single-crystal surfaces, where the cations remain in their hydration shell.^9^

Several models have been proposed for the EDL associated with mineral aqueous interfaces,^5,6^ mostly describing the contact layer in terms of outer sphere complexes of counterions, contained within a solvation shell. The Gouy–Chapman–Stern model is in this respect the most invoked picture of an electrified interface.^6^ A variety of specific models have been evaluated using a range of indirect methods such as capacitance measurements.^10^ For electrified metal oxide interfaces, modeling has been carried out for both inner sphere coordination (Grahame layer), with intimate bonding of ions to the substrate, as well as for outer sphere coordination.^11,12^

In recent years surface structure measurements have been extended from ultrahigh vacuum (UHV) environments to interfaces with aqueous solutions. TiO_2_ is the most investigated substrate material for metal-oxide electrolyte interfaces, as it is the prototype photocatalyst and photoelectrochemical system. This coincides with the role of rutile TiO_2_(110) as the model metal-oxide substrate in UHV surface science.^13,14^ Aqueous interface measurements involving oxide substrates have employed techniques including photoelectron spectroscopy,^15^ nonlinear optical methods,^6^ scanning probe microscopy (STM),^16^ and surface X-ray diffraction (SXRD).^17^ These have not yet yielded a definitive atomic-level picture of the EDL for oxide-aqueous interfaces. Nevertheless, previous studies have highlighted the stability of oxide interfaces over a range of pH,^17−19^ and in some cases with carboxylate molecules in the contact layer.^20−22^

**Figure 1:**
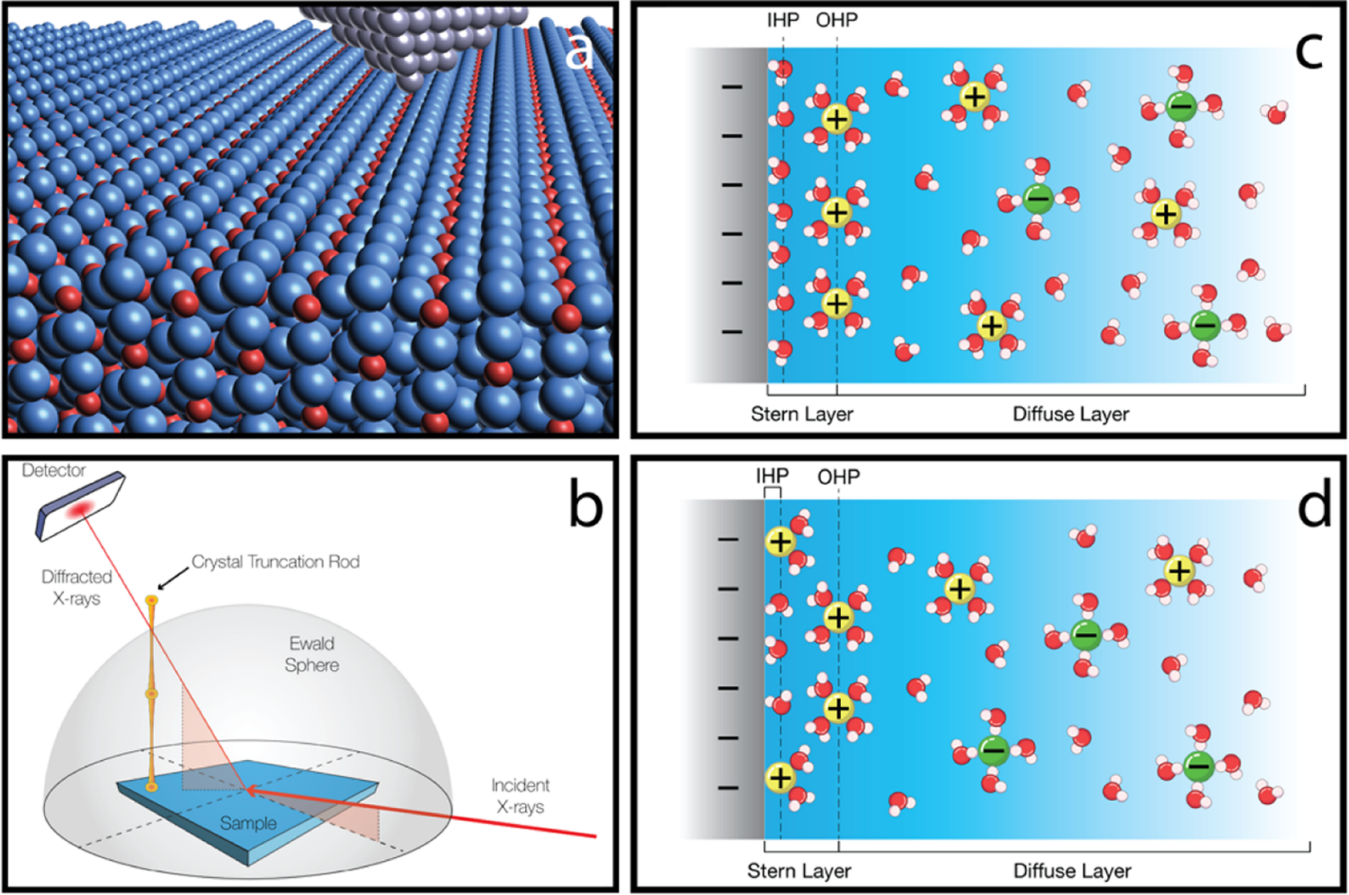
Schematic of the strategy employed to determine the electrical double-layer structure. STM imaging to determine surface order (a), with surface X-ray diffraction to determine the quantitative structure (b). Schematic of different scenarios for the arrangement of ions at an electrified interface with a high pH electrolyte, with outer sphere coordination of solvated ions in the outer Helmholtz (OHP) (c), and inner sphere coordination of ions in the inner Helmholtz plane (IHP) (d), with a diffuse outer layer in both cases.

SXRD represents a powerful method to probe the contact layer and near interface in atomic level detail, as demonstrated in previous work where chemically specific aqueous-interface Ti–O distances are observed.^20,23−25^ Here we simplify the SXRD-studied oxide-electrolyte interfaces as much as possible by employing atomically resolved STM and SXRD-characterized clean TiO_2_(110) substrates before contact with the electrolytes. Moreover, we use HCl and NaOH electrolytes to study interfaces at pH 1 and pH 13, which are below and above the PZC of 5.5.^9^ We find that both interfaces are stable and contain inner sphere ions, highlighting a difference in behavior for metal and oxide-electrified interfaces. A schematic of our approach to this investigation is summarized in Figure 1.

## 2 Experimental Details

### 2.1 Liquid Cell Scanning Tunneling Microscopy

Substrate preparation for the liquid cell STM measurements was performed using an Omicron AFM/STM instrument with a base pressure of 1 × 10^–10^ mbar. TiO_2_(110) single crystals (*Pi Kem*) were mounted onto a Ta plate with Ta strips. The sample was prepared in UHV with cycles of Ar^+^ sputtering (*P*_Ar_ = 5 × 10^–5^ mbar, 1 keV, 10 mA cm^–2^, 10 min) and annealing (*T* < 1023 K, 10 min). Characterization employed room temperature scanning tunneling microscopy (STM), low energy electron diffraction (LEED) and Auger electron spectroscopy (AES) to ensure an ordered and contaminant-free surface for the *in situ* liquid cell STM measurements. After UHV preparation and characterization, the samples were transferred to a peripheral UHV chamber and vented to N_2_. Subsequently, under a flow of N_2_, the sample was unmounted from the Ta plate and transferred to a beaker containing the experiment-specific electrolyte solution. Next, the sample was transferred to an Agilent 5500 SPM liquid cell STM that was then filled with the aqueous solution. The STM-filled state images presented were collected under constant current conditions with a sample bias in the range of −0.1 to −0.6 V. Scan angles were routinely rotated by 90° to ensure that the structures observed were not artifacts. Commercial, wax-coated 0.25 mm diameter, Pt/Ir STM tips with 10 pA maximum leakage current (Keysight Technologies) were used for the liquid cell STM measurements. The aqueous solutions (0.1 M NaOH and 0.1 M HCl) were prepared by dissolving NaOH pellets (Fischer Scientific) and diluting aqueous HCl (Sigma-Aldrich) in ultrapure water (18 MΩ cm).

### 2.2 Surface X-ray Diffraction (SXRD)

All SXRD measurements were performed at room temperature using Diamond Light Source beamline I07/surface and interface diffraction.^26^ Experimental Hutch 1 (EH 1) was employed with data acquisition performed using a (2 + 3) diffractometer and a PILATUS 100 K area pixel detector. Substrate preparation was performed on an Omicron VT STM instrument with a base pressure of 1 × 10^–10^ mbar. 8 ×10 mm^2^ TiO_2_(110) single crystals (Matek) were mounted onto a Ta plate with Ta strips. The sample was prepared in UHV with cycles of Ar^+^ sputtering (*P*_Ar_ = 6 × 10^–6^ mbar, 1 keV, 2 mA cm^–2^, 10 min) and annealing (*T* < 1023 K, 10 min) and characterized with room temperature STM, LEED and X-ray photoelectron spectroscopy (XPS) to ensure an ordered and contaminant free surface for SXRD measurements. An STM image recorded following sample preparation is shown in Figure S1 along with a structural model of the surface.

After UHV preparation and characterization, the samples were transferred, in UHV, to a baby chamber with a base pressure of 1 × 10^–9^ mbar. The baby chamber was subsequently mounted onto the diffractometer in EH 1. SXRD measurements of the clean surfaces involved the collection of 15 CTRs (2502 nonequivalent reflections). SXRD measurements of the TiO_2_(110)/electrolyte interface were then performed, which involved venting the baby chamber to N_2_ and using a droplet cell described elsewhere^27^ to form an electrolyte droplet on the surface. The X-ray beam was constrained to be always smaller than the 4 mm diameter droplet. Before use, the cell was flushed with several liters of ultrapure water to ensure that the syringe flow ran smoothly. The system was then flushed with several liters of the desired electrolyte over several hours. This was typically carried out while measuring the UHV substrate and was ready for use the moment the chamber was vented with N_2_.

Fifteen CTRs (2465 structure factors) were collected for the TiO_2_(110)/0.1 M NaOH interface and 9 CTRs (1956 structure factors) were collected for the TiO_2_(110)/0.1 M HCl interface. The SXRD data were acquired in stationary scanning mode^28^ at an incident photon energy of 17.7 keV (TiO_2_(110)/0.1 M NaOH measurements) or 23 keV (TiO_2_(110)/0.1 M HCl measurements) at an incidence angle of 1°. The goodness of fit of the SXRD model is given via the reduced χ^2^^29−31^ Data from the pioneering work of Zhang et al.^17^ for RbCl/RbOH on TiO_2_(110) at pH 12 show more structure in the anti-Bragg regions than observed here for 0.1 M HCl. This could be due to the use of a thin film cell (which is incompatible with the sample characterization methods employed here) rather than a droplet and/or the use of less extreme conditions.

Reference reflections were acquired to ensure that the surface structure did not change during the course of the experiment. The low scattering factor of H atoms prevents their experimental identification in the structure. SXRD modeling was performed using the ROD^29^ software with an orthorhombic surface cell of dimensions *a* = 6.497 Å, *b* = 2.958 Å, *c* = 6.497 Å and α = γ = β = 90°. We interpret our results in light of previous SXRD studies, where characteristic Ti–O distances are found for UHV prepared TiO_2_-aqueous interfaces: Ti–OH [1.94 Å (110)^23^, (011),^24^ (101)];^25^ Ti-carboxylate 2.1 Å (110);^20^ Ti–H_2_O (101) 2.2 Å.^25^ These distances are similar to those observed for the same species formed in UHV: Ti–OH [1.89 Å (110)^32^]; Ti–H_2_O [2.2 Å (110)^33^]; Ti-carboxylate [2.1 Å (110)^34,35^]. The technique is also capable of identifying structural variations due to different substrate preparation.^17,36^

## 3 Computational Details

The TiO_2_ surface model used in this study is analogous to that used in our previous work.^23^ It employed a 4 × 2 unit cell with four O–Ti–O layers (64 formula units in total), keeping the bottom layers fixed at bulk positions. Note that this does not imply that a 4 × 2 symmetry is present, but simply that a 4 × 2 unit cell is sufficient to describe all the important interactions. Periodic images were separated perpendicular to the surface by ca. 15 Å. Experimental lattice constants were used, resulting in lattice dimensions of 11.836 by 12.9938 Å. The role of Ti interstitials was investigated by adding 2 Ti atoms (corresponding to 0.25 ML) to the second subsurface layer and removing 4 protons from the interface for charge compensation. This concentration and positioning of the interstitials were found to be effective in explaining results for the TiO_2_(110)/water interface.^23^ All calculations were performed using the Vienna ab initio simulation package^37,38^ (VASP, version 5.4) and standard projector augmented-wave potentials. We used two different density functional approaches, (i) optB86b-vdW^39,40^ and (ii) PBE^41^ together with a Hubbard *U* correction^42^ of 4.2 eV for the Ti 3d orbitals and the D3 dispersion correction.^43,44^ As the latter is expected to give more reliable results for interfaces with Ti interstitials present, the results in Figure 3 and the corresponding discussion in the main text are associated with these PBE + *U* + D3 calculations. Spin-polarized calculations were performed for structures containing Ti interstitials. Calculations for the UHV interface used a plane-wave kinetic energy cutoff of 600 eV, and convergence criteria for SCF and forces were set to 10^–6^ eV and 10^–2^ eV/Å, respectively.

AIMD simulations were performed with a Nosé–Hoover thermostat at 300 K, deuterium masses for hydrogen and a time step of 1 fs. 72 water molecules were added on top of the UHV interface structures, leading to a ca. 12 Å high water phase. The initial water structure was obtained by equilibrating a random arrangement of water molecules on a TiO_2_(110) surface without Ti interstitials and NaOH or HCl for 20 ps. The water molecules were then transferred to the corresponding interface and equilibrated for 5 ps with all other atoms fixed before the production runs (35 ps) were performed. The cutoff and SCF convergence criteria were reduced to 400 and 10^–4^ eV, respectively. Sampling the Brillouin zone was performed using the Γ point only for the 4 × 2 unit cell in both the UHV interface calculations and the AIMD simulations. Structural information (i.e., the vertical and lateral positions and radial distribution functions, as shown in Figure 3 in the main text) was obtained by averaging over snapshots taken at 500 fs intervals. Bader charges^45^ were calculated for a snapshot taken after 30 ps using a grid-based approach.^46^ To ensure the reproducibility of the computational results reported herein, input files for the key simulations reported in the main text are provided as a separate set of files in the SI.

## 4 Results and Discussion

After showing that the phase-boundaries of the UHV-prepared surfaces with electrolytes are stable using scanning tunneling microscopy (see Figures S1 and S2), SXRD data were recorded in the form of crystal truncation rods (CTRs)^47^ to determine the quantitative interface structure. Each CTR is recorded by measuring the scattered X-ray intensity with the momentum transfer along the reciprocal lattice vector *l* for a particular value of [*hk*]. The intensities between the bulk diffraction peaks, in the so-called anti-Bragg regions, contain information about surface and interface structure. This arises from symmetry breaking at the surface, such that scattering is no longer isotropic. Diffuse intensity then appears perpendicular to the surface.

**Figure 2:**
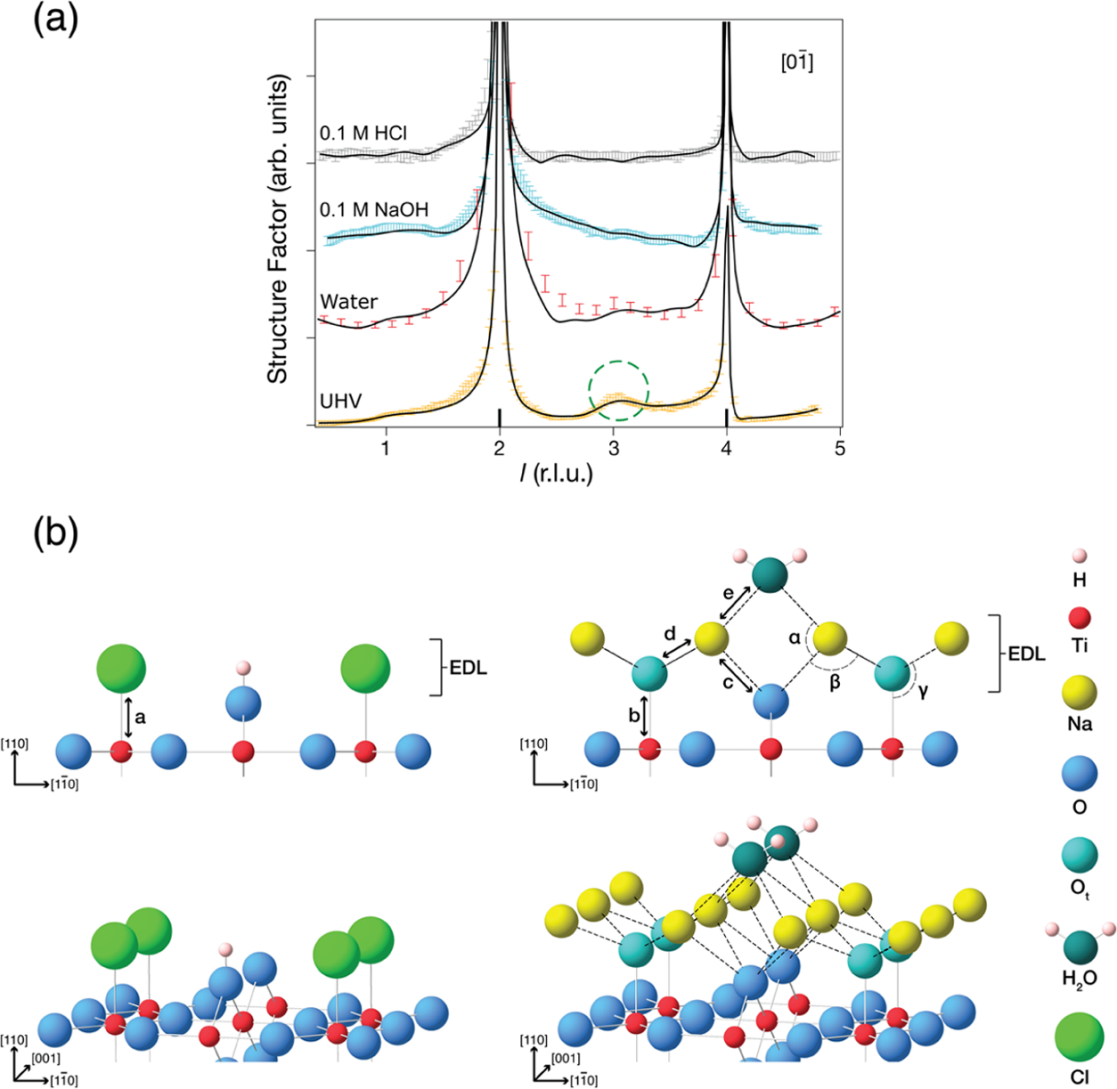
Surface X-ray diffraction results and the corresponding best-fit models. (a) CTRs [0 1̅*l*] for TiO_2_(110) in UHV (orange) and, submerged in H_2_O_(l)_ (red),^23^ 0.1 M NaOH (blue) and 0.1 M HCl (gray), shown on a linear scale and offset for clarity. The black lines are the best fits to the data. Bars on the *x*-axis indicate the position of bulk Bragg reflections. The green circle represents an “anti-Bragg” modulation that is suppressed upon liquid exposure. This is attributed to surface Ti atoms adopting a more bulk-like position. The TiO_2_(110)/0.1 M NaOH and TiO_2_(110)/0.1 M HCl CTRs are strikingly different for 2 < *l* < 4. This suggests a significant effect of pH and/or electrolyte on the behavior of surface atoms and surface adsorption. (b) Ball and stick illustration depicting the TiO_2_(110)/0.1 M HCl (left) and 0.1 M NaOH (right) interface structures obtained from SXRD. Side and tilted views are shown of the interface structure. H atom positions are guided by theoretical calculations. The relaxation of substrate atoms is shown in Table S1, with the full structure files provided as separate files in the SI. The distance and angle values in (b) are *a* = 2.24 ± 0.02 Å; *b* = 2.03 ± 0.02 Å; *c* = 2.77 ± 0.01 Å; *d* = 2.40 ± 0.01 Å; *e* = 2.80 ± 0.02 Å; α = 77 ± 1°; β = 80 ± 1° and γ = 113 ± 1°.

Data for the [01̅*l*] CTR from as-prepared TiO_2_(110) and the H_2_O, 0.1 M NaOH, and 0.1 M HCl interfaces with TiO_2_(110) are shown in Figure 2a, along with the corresponding best fits to the SXRD data. Data analysis used the ß-roughness method,^47^ which also takes defects into account, with this parameter increasing from 0.02 for the clean surface to 0.17 and 0.19 for the NaOH and HCl interfaces, respectively. These values for the electrolyte interfaces are similar to those obtained for the interface with water.^23^ The full data sets and their fits are shown in Figure S3, which have a χ^2^ (see Supporting Information (SI)) of 1.12 (clean surface), 1.7 (H_2_O),^23^ 1.15 (NaOH) and 1.03 (HCl) for the best fit atomic positions shown in Table S1. Selected CTRs are compared to the best fit to different possible models of the interfaces in Figures S4 and S5. For the TiO_2_(110)/HCl [20] rod, detector images for selected *L* values are shown in Figure S4c. The labeling of the atoms is shown in Figures S6 and S7, which does not include the H atoms, to which SXRD is insensitive. Corresponding substrate atom displacements are shown in Table S1. The atomic displacements for the as-prepared surface are essentially identical to those found previously.^23,48^ Surface atoms displace away from the bulk, a phenomenon that has previously been observed on TiO_2_(110)^23,48^ and other TiO_2_ surfaces such as anatase TiO_2_(101).^49^

Turning first to the interface of TiO_2_(110) with 0.1 M NaOH, Figure S8 illustrates the potential adsorption sites of Na on R_110_ when an O species is bound to Ti_5c_. This will most likely be a terminal O species (O_t_), terminal OH (OH_t_), or an H_2_O molecule. The optimized structure of the TiO_2_(110)/0.1 M NaOH interface indicates that Na adsorbs at the tetradentate site (between two O_2c_ and two terminal O species bound to Ti_5c_), with other models in Figure S8 giving χ^2^ > 1.5. If Na is replaced with oxygen in the best fit site, χ^2^ increases from 0.9 to 1.35 (*R*-factors: 16 to 18.6%). The best-fit model is shown in Figures 2(b) and S6, where the Ti_5c_–O distance is 2.03 ± 0.01 Å, with a corresponding O–Na bond length of 2.40 ± 0.01 Å and an O_2c_–Na bond length of 2.77 ± 0.01 Å. Above the second layer tetra-dentate Na there is a layer of oxygen atoms (O, OH^–^, or H_2_O) residing in tetradentate sites between four Na atoms at 2.80 ± 0.01 Å. There is a full occupation of these Na and O adsorbates, providing a 6-fold O-coordinated Na. A plot of χ^2^ against Na occupation in the best-fit model is shown in Figure S9. The O–Na bond lengths are in line with those reported in several environments, 2.3–2.9 Å.^50−52^ A similar bonding site of K adsorbed on hydroxylated TiO_2_(110) was found in a UHV study, differing from the interface structure investigated here in that only one terminal O species is involved.^53^ Multiple sites were considered for Rb^+^ at a RbCl/RbOH pH 12 aqueous interface with TiO_2_(110), with a preference for a tetradentate site at 0.4 ML coverage,^17^ a difference which likely arises from the sample preparation employed. In the Ti_5c_–O–Na moiety, O will be negatively charged, and Na positively charged, providing an ultracompact double layer at the interface. This inner Helmholtz plane is a manifestation of a Grahame layer, distinguished by the inner sphere or direct coordination of the counterions to the substrate. The EDL is indicated in Figures 2(b) and S6. Experimentally determined atomic positions for both interfaces are shown in separate files in the SI.

The best-fit SXRD model of the TiO_2_(110)/0.1 M HCl interface suggests full occupation of Cl adsorbates at the Ti_5c_ site (see Figures 2(b) and S7), with a Ti_5c_–Cl bond length of 2.24 ± 0.02 Å (see Figure S10 for the variation of χ^2^ with Ti_5c_–Cl bond length and Figure S11 for the variation with Cl coverage). This adsorption site was also identified following the adsorption of Cl in UHV,^54,55^ with a calculated Ti_5c_–Cl bond length of 2.2 Å.^55^ More generally, Ti–Cl bond lengths are reported between 2.2 and 2.4 Å.^56−62^ Alternative models, including the adsorption of O (OH_t_ or H_2_O) species at the Ti_5c_ site were also tested. However, these resulted in significantly higher χ^2^ values of 1.68 and 1.39, respectively. The presence of a water layer above the adsorbed Cl species was also probed to investigate the existence of H_2_O molecules either hydrogen bonded to O_2c_ or forming an electrostatic interaction with the adsorbed Cl atoms, although an ordered overlayer was not found. Recent ambient pressure X-ray photoelectron spectroscopy measurements also identify Cl atoms at the interface of TiO_2_(110) with 0.1 M HCl (see Figure S12).^63^

Density functional theory geometry optimizations and ab initio molecular dynamics (AIMD) simulations are employed to explain the stability of the interfaces. First, we investigated the UHV/NaOH/TiO_2_ and UHV/HCl/TiO_2_ interfaces as reference points for the later solid/liquid interface calculations. We investigated Na coverages from 0.25 ML (1 Monolayer corresponds to the number of Ti_5c_ atoms) up to 2 ML (NaOH) and Cl coverages from 0.125 up to 1 ML (HCl). The lowest energy high-coverage adsorption structures are in reasonable agreement with the SXRD experiments, with Cl on Ti_5c_ and tetradentate bonding of Na to O_br_/O_t_.

We performed AIMD simulations to evaluate the stability of high-coverage HCl and NaOH layers at the solid/liquid electrolyte interface. Some ions leave the interface within the first 5 ps of the simulation. Two out of eight Cl ions and four out of 16 Na ions move into the liquid water. No diffusion back toward the interface occurred within the 30 ps runs. For the TiO_2_(110)/NaOH interface, we tested a different initial interface structure consisting of terminal O and H_2_O on top of Na, instead of terminal OH with OH on top of Na. This leads to a slightly more stable interface, but two Na ions still move away from the interface. Since Ti interstitials are important for the stability of the (2 × 1)–OH overlayer^23^ we included them in additional AIMD simulations. The Ti interstitials increase the stability of the HCl and NaOH interfaces, so that all Na and Cl ions remain at the interface. Analysis of Bader charges (see Table S2) reveals that the charge of Cl ions strongly depends on the presence of Ti interstitials (−0.55, compared to −0.68 without interstitials). The charge of Na ions, on the other hand, is nearly unaffected (0.86, compared to 0.85 without interstitials). Although interstitials are needed for the calculations, they are not evidenced in SXRD results of the clean (110) and water covered surface published earlier^23^ nor in data obtained from the electrolyte interfaces, presumably because they are not long-range ordered. Their presence will be taken into account by the β-roughness parameter.

**Figure 3:**
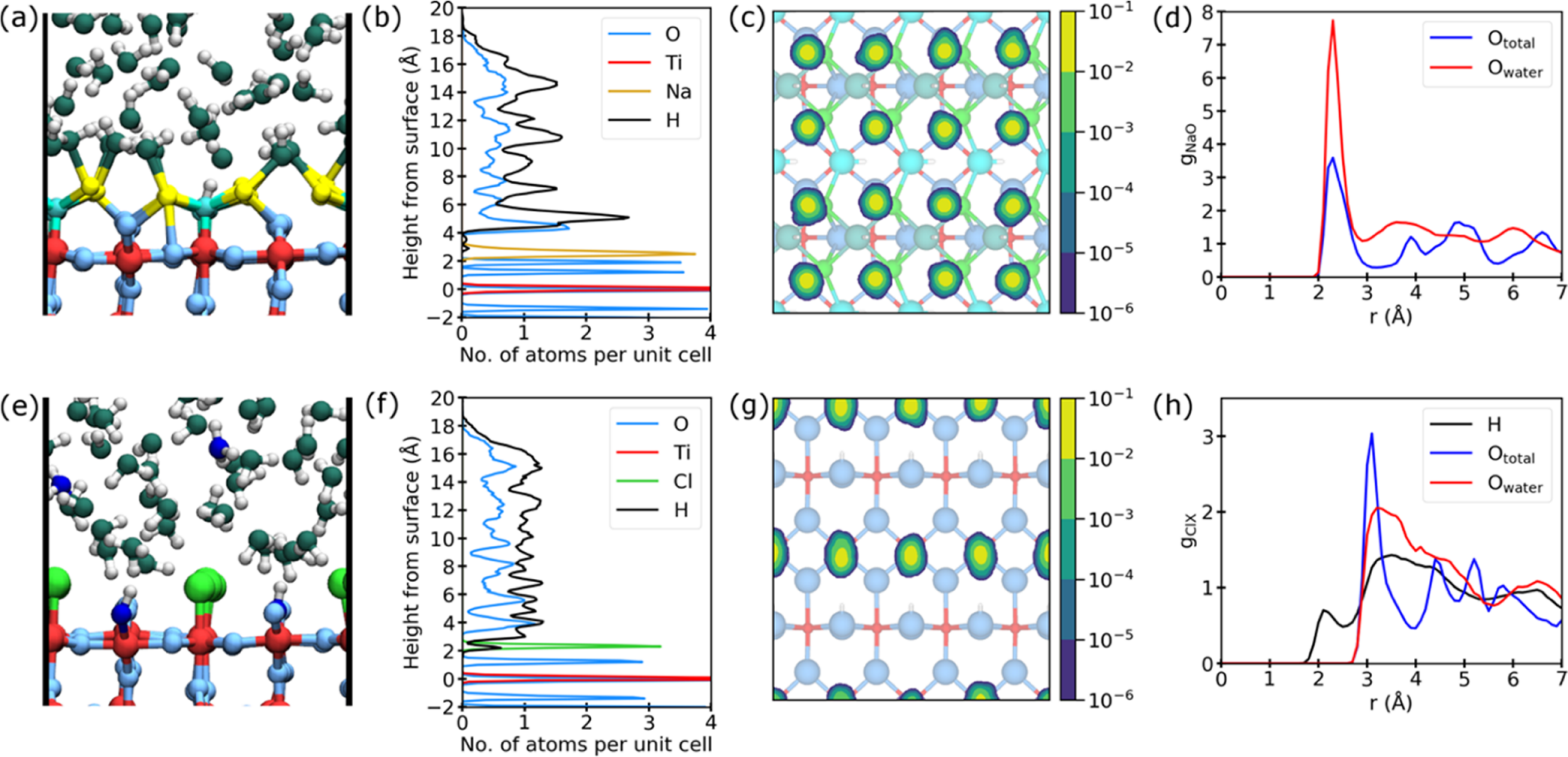
Results from AIMD simulations of the TiO_2_(110)/NaOH interface (a–d) and the TiO_2_(110)/HCl interface (e–h). (a, e) Snapshots after 30 ps of simulation (color code as in Figure 2, with H_3_O^+^ and OH^+^ (O_br_ + H^+^) shown in dark blue). (b, f) Time-averaged distribution of atoms above the EDLs along the surface normal (the [110] direction). (c, g) Time-averaged distribution of Na and Cl ions within the (110) plane. (d, h) Radial distribution functions *g_XY_*. The distribution functions are individually normalized, so for example, unity for *g*_Cl-Owater_ corresponds to the average density of O_water_ in the simulation.

Due to the choice of the initial interface structure (with terminal O instead of terminal OH), charge neutrality dictates that the O species above Na are exclusively water molecules at the start of the AIMD calculations. During the AIMD simulation one proton transferred from a water molecule to a terminal O. Since the OH_t_–Ti distance is slightly larger than the O_t_–Ti distance, the difference in experimental and calculated O_t_–Ti distances (2.03 vs 1.81 Å) may indicate the presence of OH_t_ at the experimental interface. The stabilities of the AIMD solutions for the interfaces point to their overall charge neutrality. There is a slight imbalance in the charges of the double layers which is compensated by charges associated with water and ions on the solution side. The contribution from charges on O and H in water of the first layer above each double layer is included in Table S2. The structural details obtained from calculations for the substrates that contain interstitials are shown in Figure 3. The majority of Na ions, as well as all Cl ions exhibit very limited mobility, both vertically (panels a,b,e and f) and laterally (panels c and g). At both interfaces, the first water layer shows the expected orientation. H atoms point toward the Cl interface, while they point away from the Na interface (Figure 3(a,e)). In both cases there is considerable mobility of water molecules, with a variation in coordination. This compares with our experimental time-averaged tetradentate coordination. The extent of the ion and molecule mobilities is evidenced in the animations of SI Movies 1 and 2.

The first peak in the Na–O radial distribution function at the interface is located at about 2.3 Å (see Figure 3(d)), which is in reasonable agreement with the average Na–O distance of 2.6 Å found in the SXRD experiment. Perfect agreement between experiment and theory is not expected because of the approximations involved in the calculations. Principally, these are associated with the number of substrate layers included, the density functional theory (DFT) functional employed, and the movement of atoms in the AIMD simulation. The calculated coordination number of Na of 6.8 ± 0.7 compares well with the experimental value of 6.0. Interestingly, both these values are higher than the Na–O_water_ coordination number in NaOH solution (5.3),^64^ which may explain the preference for inner-sphere coordination. A summary of the comparison between average bond lengths from AIMD simulations and the corresponding SXRD values is shown in Table S3. In general, there is good agreement, with the shorter distance predicted by DFT for Ti_5c_–O_t_ and O_t_–Na likely due to a degree of protonation of terminal O atoms in the experiment.

The calculated Ti–Cl distance at the TiO_2_(110)/HCl interface of 2.37 ± 0.15 Å is also in reasonable agreement with experiment (2.24 ± 0.01 Å). At pH 1 the substrate is thought to be covered by positively charged H^+^. Our calculations for the TiO_2_(110)/HCl interface start with the substrate containing bridging hydroxyls, with H on bridging O (O_br_) (O 1 in Figure S7). In the absence of Ti interstitials, all O_br_ are protonated initially. To remain charge-neutral in the simulations with Ti interstitials, half of the H^+^ is removed from the initial structure. During the MD simulations, around 50% of the H^+^ are transferred to water molecules. This is independent of the presence of Ti interstitials. Hence, O_br_H is evidenced in the calculations. Moreover, H_3_O^+^ is also found at the interface. Both of these positive species will provide the counter charge to negative Cl in another ultracompact double layer. Examples of their positions are shown in Figure 3, with O_br_H and the EDL position also included with the SXRD model in Figures 2(b) and S7. DFT calculations^65^ suggest that the energy difference between the adsorbed chloride and chloride in solution is rather small but favors the adsorbed species. At higher coverages, this becomes slightly less stable due to lateral interactions, but our calculations show that this is compensated by a stronger interaction with a surface when the selvedge contains Ti interstitials. In our AIMD simulation, which simply samples the lowest energy solutions, this is evidenced by a reduction in the adsorbate desorption rate.

The two double layers have widths of about 1 Å, with associated electric fields that will significantly affect the interface’s chemical properties. For instance, the energetics of the water oxidation reaction over TiO_2_(110) are expected to be influenced by the EDL potential.^66^ Many other applications will be influenced by this high electric field gradient, with the ion and molecule mobilities (SI Movies 1 and 2) pointing to reactants access to the interface. Moreover, the strong binding of ions to TiO_2_ evidenced here also suggests a role in the binding of ions associated with their removal from solution, for example in water treatment applications.^67^

The first peaks in the Cl–H and Cl–O_water_ radial distribution function (2.1 and 3.2 Å, respectively, see Figure 3(h)) are also in good agreement with the corresponding values for an HCl solution (2 and 3 Å, respectively).^68^ However, the coordination environment of Cl with water molecules is not as clearly defined, with a broad distribution of distances. This is consistent with the lack of water ordering above Cl observed in the SXRD experiment. Taking a cutoff value of 4 Å (based on the first minimum in the radial distribution function), the number of coordinated water molecules in the first solvation shell (3.4 ± 1) is lower than for an HCl solution (4.4), but this is compensated by the bond to Ti. Similar to the NaOH interface, there is an increased density of water molecules in the first water layer. Along with the preferred orientation of water molecules found at each interface, this could be part of the structure associated with the outer Helmholtz planes.^5^ Obtaining further details of these structures and those of the diffuse layers (see Figure 1(d)) is beyond the scope of the current work.

## 5 Conclusions

We report the formation of ultracompact electrical double layers at the interfaces of TiO_2_(110) with 0.1 M HCl and 0.1 M NaOH. To the best of our knowledge, this is the first quantitative structure determination of metal oxide electrified interfaces using a surface science approach. This involved atomic level characterization of the substrate, allowing us to determine single interface structures for both acidic and alkaline environments. Their formation is explained by AIMD simulations. The structures obtained from surface X-ray diffraction contain nonsolvated Cl and Na in the contact layers, with saturation coverage. A comparison with the results of ab initio molecular dynamics calculations points to the formation of double layers with a width in the region of 1 Å. These results are consistent with a model of the electrical double layer at metal oxide electrified interfaces that involves specifically adsorbed ions. They represent previously unanticipated pH-dependent structures with extremely high electric fields (ca. 10^10^ V m^–1^), suggesting that they will play a pivotal role in determining the reactivity of the interface. The observation of inner sphere ions for both acidic and alkaline environments points to a different behavior from that of metal substrates. Hence, these results have a broad significance for the field of metal oxide aqueous interfaces, including those involved in photochemistry, mineralogy, and colloid chemistry. For photochemistry applications there will also be a considerable influence on the electron dynamics, for which theoretical calculations now have robust and accurate structural data.

## Supporting Information Available

Ab initio molecular dynamics simulation of the TiO_2_(110)/NaOH interface (MP4)
Ab initio molecular dynamics simulation of the TiO_2_(110)/HCl interface (MP4)
STM results for TiO_2_(110) in UHV and under 0.1 M HCl and 0.1 M NaOH; comparison of SXRD data for TiO_2_(110) in UHV, and covered with ultrapure water, 0.1 M HCl and 0.1 M NaOH; comparison of SXRD data with different models of the TiO_2_(110) interfaces with 0.1 M HCl and 0.1 M NaOH; best fit models of the interfaces highlighting the position of the electrical double layer; χ^2^ variation with Na occupancy for the TiO_2_(110)/0.1 M NaOH interface; χ^2^ variation with Ti_5c_–Cl bond length and Cl occupancy at the TiO_2_(110) interface with 0.1 M HCl. Ambient pressure XPS spectra evidencing Cl at the interface of TiO_2_(110) with 0.1 M HCl; tabulated values of the displacements in the substrate associated with formation of electrolyte interfaces, and Bader charges associated with the interfaces; bond length comparisons between DFT and SXRD and stills from movies of the MD simulations of the interfaces (PDF)
Input parameters for the simulations and experimentally determined atomic positions for the TiO_2_(110)/NaOH and TiO_2_(110)/HCl interfaces (ZIP)
