# Supplementary material for: Ultracompact Electrical Double Layers at TiO2(110) Electrified Interfaces
Source: J Am Chem Soc. 2024 Nov 25;146(49):33443–51. doi: 10.1021/jacs.4c09911 (PMC11638939; doi:10.1021/jacs.4c09911)
Supplement: Supplementary file 3 — ja4c09911_si_003.pdf [file ja4c09911_si_003.pdf]

Supplementary Information

Ultra-Compact Electrical Double Layers at TiO_2(110) Electrified Interfaces

Immad M. Nadeem[1,2], Christopher Penschke[3]†, Ji Chen[3]‡, Xavier Torrelles[4], Axel Wilson[1,2], Hadeel Hussain[2], Gregory Cabailh[5], Oier Bikondoa[6,7], Jameel Imran[1], Christopher Nicklin[2], Robert Lindsay[8,9], Jörg Zegenhagen[2], Matthew O. Blunt[1], Angelos Michaelides[3]§ and Geoff Thornton[1*]

[1] London Centre for Nanotechnology and Department of Chemistry, University College London; 20 Gordon Street, London WC1H 0AJ, UK

[2] Diamond Light Source Ltd; Harwell Science and Innovation Campus, Didcot, Oxfordshire OX11 0DE, UK

[3] London Centre for Nanotechnology and Department of Physics & Astronomy, University College London; 17-19 Gordon Street, London WC1H 0AH, UK

[4] Institut de Ciència de Materials de Barcelona (CSIC), Campus UAB; 08193 Bellaterra, Spain

[5] Sorbonne Université, CNRS, UMR 7588, Institut des NanoSciences de Paris; 4 Place Jussieu, F-75005 Paris, France

[6] Department of Physics, University of Warwick; Gibbet Hill Road, Coventry CV4 7AL, UK

[7] XMaS, the U.K. CRG Beamline, ESRF, The European Synchrotron; 71, Avenue des Martyrs, CS40220, F-38043 Grenoble cedex 09, France

[8] Corrosion and Protection Centre, Department of Materials, The University of Manchester; Sackville Street, Manchester M13 9PL, UK

[9] Photon Science Institute, The University of Manchester; Manchester M13 9PL, UK

*Corresponding Author. Email: g.thornton@ucl.ac.uk

Present Addresses:

† Institut für Chemie, Universität Potsdam; Karl-Liebknecht-Straße 24-25, D-14476 Potsdam-Golm, Germany

‡ School of Physics, Peking University; Beijing, 100871, China

§ Yusuf Hamied Department of Chemistry, University of Cambridge; Lensfield Road, Cambridge, CB2 1EW, UK

Supplementary Information includes:

Figs. S1, S2; STM images of $TiO_2(110)$ in UHV and under electrolytes

Figs. S3-S5; Experimental crystal truncation rods along with fits to the data for different structural models

Figs. S6, S7; Structural models of the interfaces

Fig. S8; Trial models of the $TiO_2(110)$ interface with 0.1 M NaOH

Fig. S9-S11; Variation of χ^2 with Na occupancy at the $TiO_2(110)$/0.1 M NaOH interface, and Cl occupancy and bond length at the HCl interface

Fig. S12; Ambient pressure photoemission of the $TiO_2(110)$/0.1 M HCl interface

Table S1; Experimentally derived displacements of substrate atoms away from the bulk terminated positions for clean $TiO_2(110)$ compared with those at the electrolyte interfaces

Table S2; Calculated Bader charges at the interfaces

Table S3; DFT results compared to experimental bond lengths at the electrolyte interfaces

Movies Mov 1, 2; Molecular dynamics simulations of the $TiO_2(110)$ interfaces with the electrolytes

Text files containing the experimentally determined atomic positions (.xyz) and the input parameters for the simulations

References

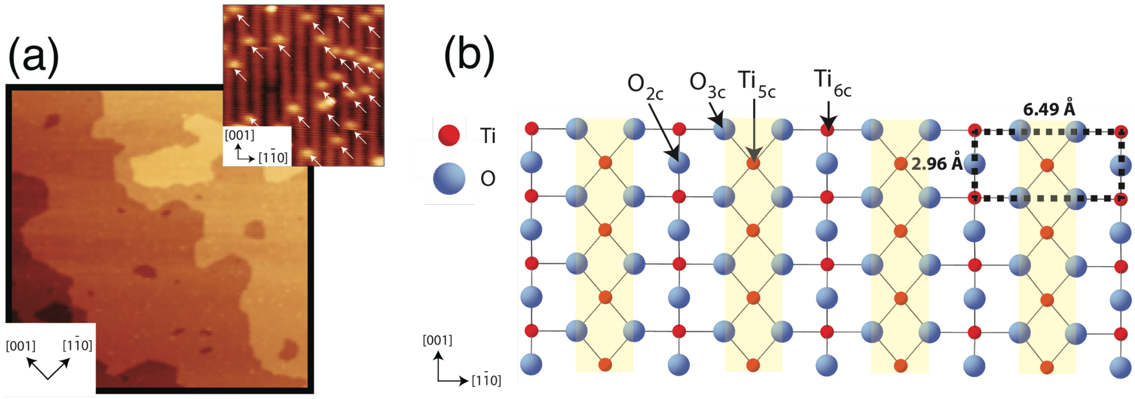

Figure S1: STM images and structural models of clean $TiO_2(110)$. (a) 300 × 300 nm^2 STM image of $TiO_2(110)$ (V_s = +1.6 V, I_t = 0.1 nA) with inset (7 × 7 nm^2 (V_s = +1.3 V I_t = 0.3 nA)) illustrating the bright and dark row features in the [001] direction. The separation of the rows is 6.5 Å[1]. The white arrows point to O_{vac} and OH_{br} sites. (b) Ball and stick model of an on-top view of the $TiO_2(110)$ surface where the light-yellow rows represent the bright rows seen in STM. The bright rows correspond to Ti_{5c} whereas the dark rows represent O_{2c} (also referred to as O_{br}).

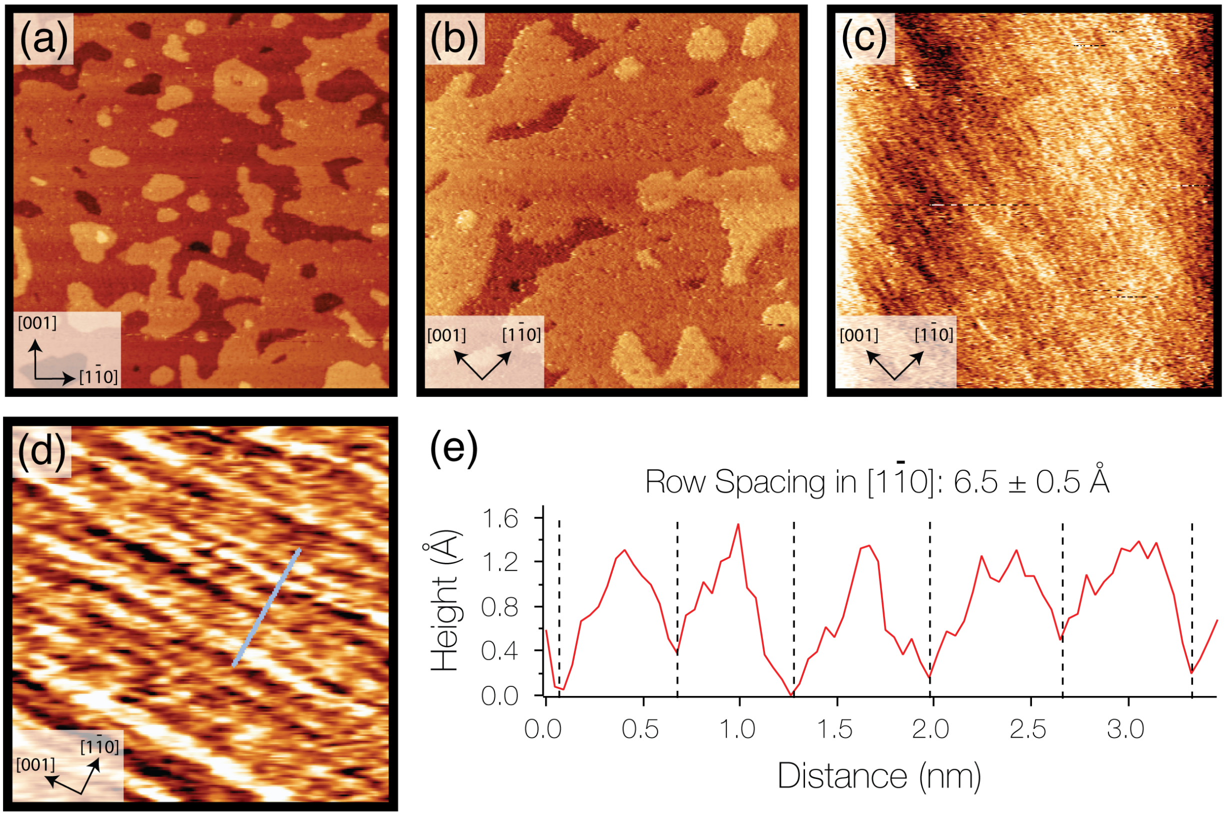

Figure S2: STM images of $TiO_2(110)$ before and after submersion in basic pH 13 and acidic pH 1 solutions. These images were found to be unchanged for up to 36 h. (a) 300 × 300 nm^2 UHV STM image of $TiO_2(110)$ (V_s = +1.6 V, I_t = 0.1 nA) prior to 0.1 M NaOH submersion. (b) 300 × 300 nm^2 liquid cell STM of $TiO_2(110)$ (V_s = -0.6 V, I_t = 3 nA) submerged in 0.1 M NaOH. (c) 25 × 25 nm^2 liquid cell STM of $TiO_2(110)$ (V_s = -0.6 V, I_t = 3 nA) submerged in 0.1 M NaOH, where [001] row structure can still be discerned. (d) This is clearer in the 7.5 × 7.5 nm^2 liquid cell STM image of $TiO_2(110)$ (V_s = -0.6 V, I_t = 1.5 nA) submerged in 0.1 M HCl (e) The line profile from (d) highlights the 6.5 Å row spacing in the [001] direction, which is that found on the clean surface (see Figure S1). The quality of the images in the electrolyte solutions is degraded compared to those recorded in UHV (see Figure S1) likely because of increased drift and modifications to the STM tip apex. Nevertheless, the row resolution achieved at high and low pH is unprecedented as far as we are aware.

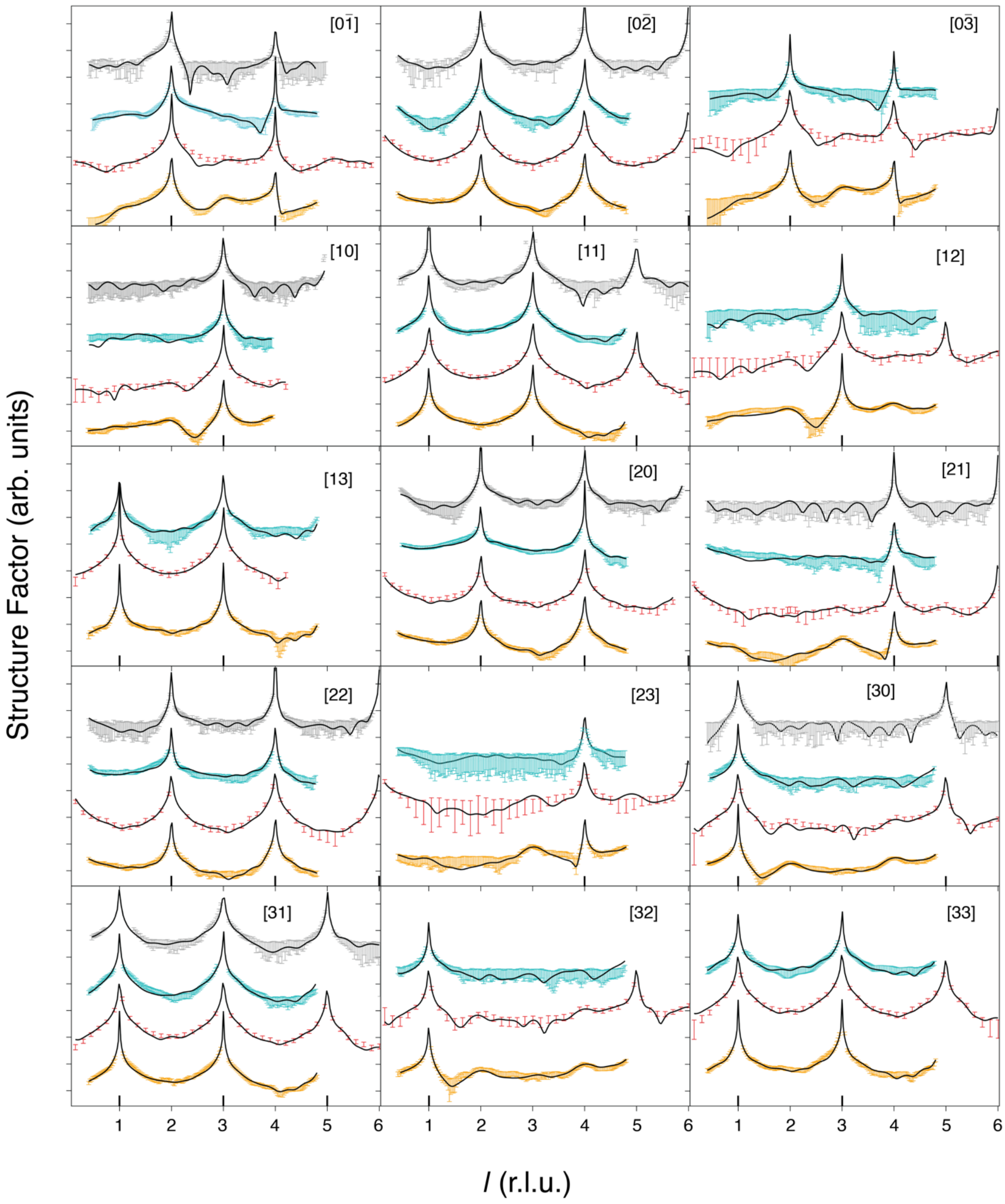

Figure S3: Comparison of experimental CTRs (color-coded error bars) and best fit (solid lines) for as-prepared TiO_2 (110) in UHV (orange), and with droplets of ultrapure H_2O (red)[1], 0.1 M NaOH (blue) and 0.1 M HCl (gray). CTRs are shown on a log scale and offset for clarity. They display significant differences in the shape on each side of the Bragg peaks for the different aqueous solutions, which are the most statistically significant in the structural analysis. An example is the $[0\bar{1}]$ rod around the $[0\bar{1}2]$ Bragg peak.

(a)

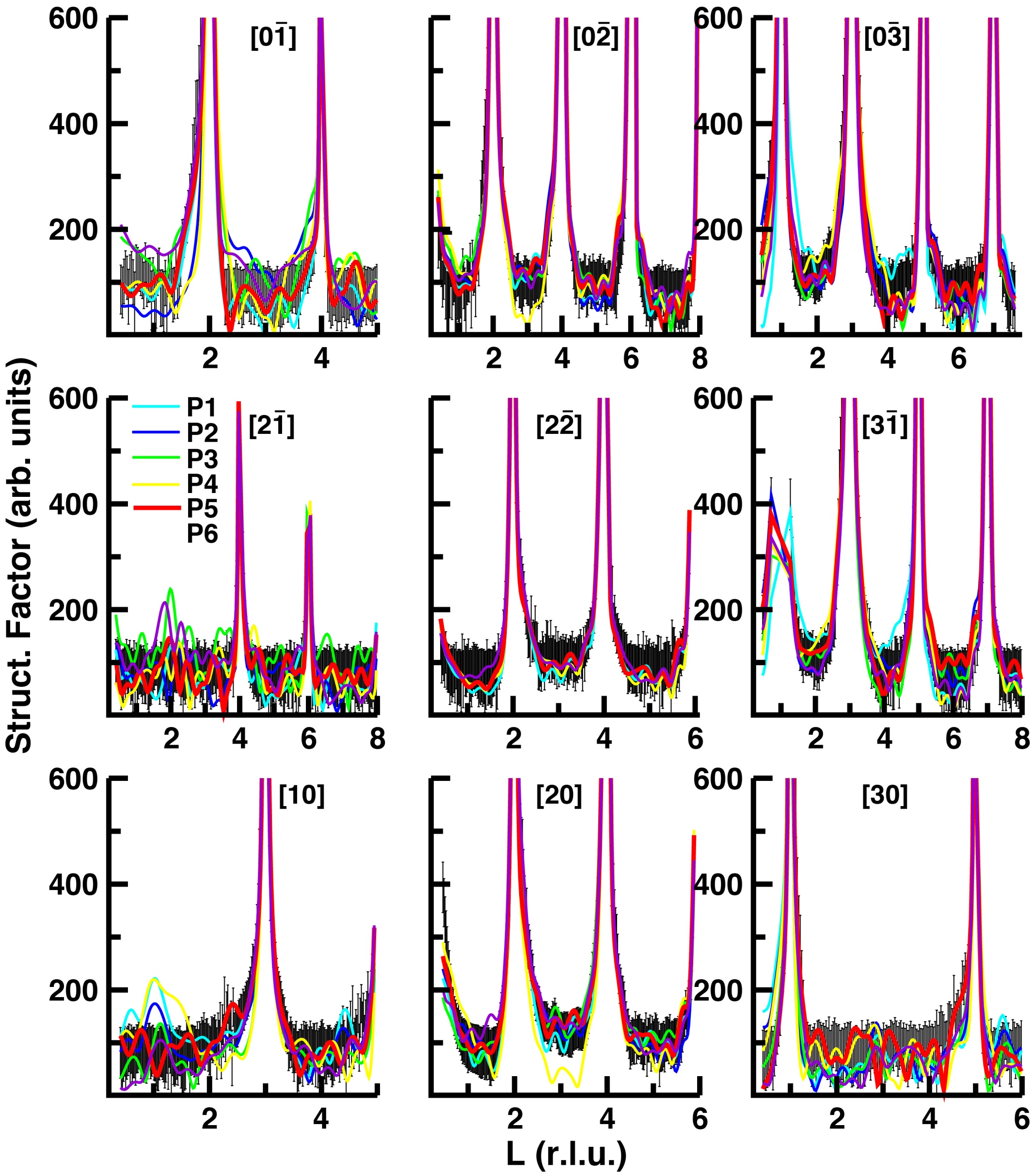
Struct. Factor (arb. units)
L (r.l.u.)
[0$\bar{1}$]
[0$\bar{2}$]
[0$\bar{3}$]
[2$\bar{1}$]
[2$\bar{2}$]
[3$\bar{1}$]
[10]
[20]
[30]
P1
P2
P3
P4
P5
P6
600
400
200
2
4
6
8

(b)

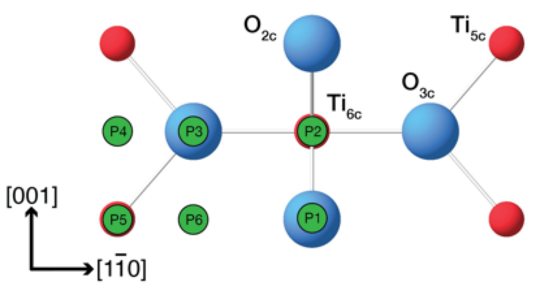
O_{2c}
Ti_{5c}
O_{3c}
Ti_{6c}
P1
P2
P3
P4
P5
P6
[001]
[1$\bar{1}$0]

(c)

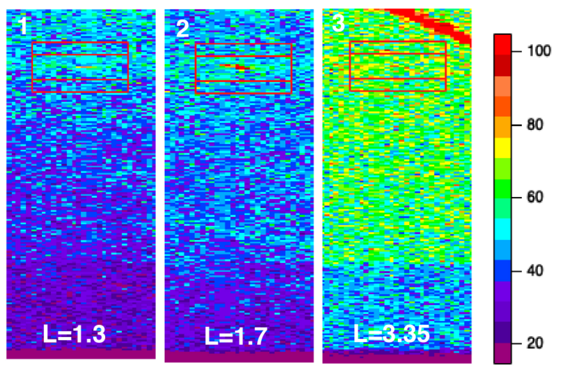
1
2
3
L=1.3
L=1.7
L=3.35
100
80
60
40
20

Figure S4: Comparison of (a) experimental CTRs (error bars) for the $TiO_2(110)$/ 0.1 M HCl interface and best fits (solid lines) for potential models P1 – P6 of the interface shown in (b). The inset in (a) identifies with a color code the fits with the models. The CTRs are shown on a linear scale and offset for clarity. χ^2 values corresponding to the best fits are: P1 1.91; P2 1.59; P3 1.48; P4 2.06; P5 1.03; P6 1.33, thus identifying the atop site P5 as the correct structural model. The χ^2 values for the other models of the HCl interface tested, while significantly higher, are relatively close to the best model compared to those for the NaOH interface. The oscillations observed in rod fits of the anti-Bragg regions arise from the large number of layers allowed to relax along the z axis, as well as the additional surface roughness at the liquid interfaces. χ^2 and the corresponding R-factor give values related to their respective average experimental errors. When $\chi^2 \approx 1$ the experimental structure factors are within uncertainties equal to the calculated values for the best fits. Here a value of $\chi^2 = 1$ corresponds to an R-factor = 23%, where the average value of the experimental errors is close to this value. (c) contains images of the 2D detector at three L values, along with an intensity color map. 2D X-ray detectors, such as that used here, require custom software to reduce the large amounts of data obtained from crystal truncation rods (CTRs) measured in stationary mode. Here, intensities along the rod are tracked by varying the L index while keeping the H and K values fixed during the measurement. Experimental intensities measured under these conditions require background subtraction. The intensity of the rod distribution along a rod (H,K,L_i), I_{CTR}, is first integrated for each L_i value by selecting a region-of interest (ROI) large enough to contain the peak area, while several ROI_{Back} can be defined to account for the background. The subtracted background is proportional to the number of ROI pixels used to integrate the peak. In the three images shown, the inner red rectangle is the ROI and the smaller rectangles shown above and below are the ROI_{back}. A higher intensity in the ROI can be seen, even where the intensity between the Bragg peaks appears flat. The red line at the top right of the image for L=3.35 arises from a powder ring. A more detailed description of the analysis protocol is given in Ref. 1.

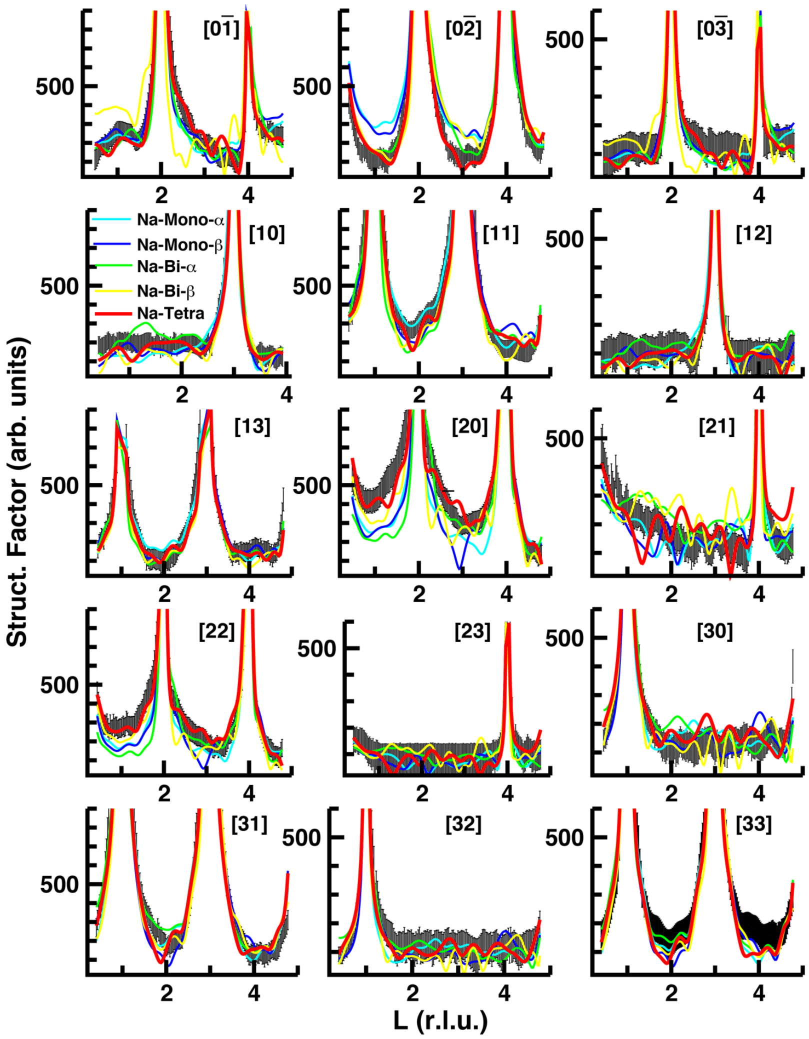

Figure S5: Comparison of (a) experimental CTRs (error bars) for the TiO_2 (110)/ 0.1 M NaOH interface and the best fits (solid lines) for five of the six potential models of the interface shown in Figure S8. The CTRs are shown on a linear scale and offset for clarity. χ^2 values for the best fits to the six models are: Na mono-dentate-α site 1.89; Na mono-dentate-β site 1.93; Na bi-dentate-α site 2.19; Na bi-dentate-β site 2.34; Na tetra-dentate 1.15; Terminal O 2.41, thus identifying the tetra-dentate site as the correct structural model. The oscillations observed in rod fits of the anti-Bragg regions arise from the large number of layers allowed to relax along the z axis, as well as the additional surface roughness at the liquid interfaces. χ^2 and the corresponding R-factor give values related to their respective average experimental errors. When $\chi^2 \approx 1$ the experimental structure factors are within uncertainties equal to the calculated values for the best fits. Here a value of $\chi^2 = 1$ corresponds to an R-factor = 16%, where the average value of the experimental errors is close to this value.

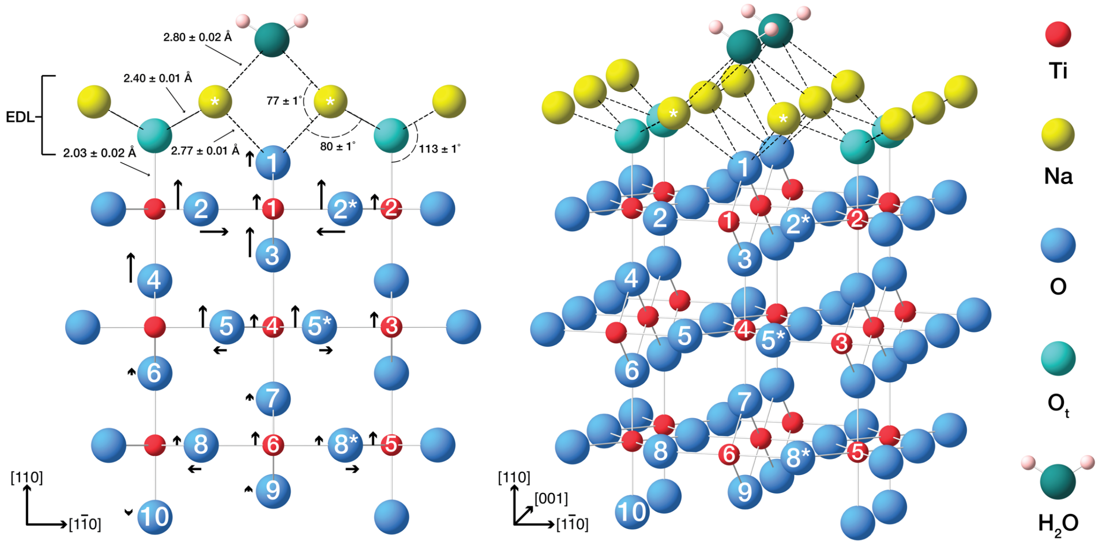

Figure S6: Ball and stick illustration depicting the TiO_2(110)/ 0.1 M NaOH interface structure obtained from SXRD. (a) Side view and (b) tilted view of the interface structure. H atom positions are guided by theoretical calculations. The black arrows represent the relative magnitude and direction of atom displacements with respect to bulk lattice positions. The indicated azimuth defines the x, y, and z directions along which the atomic coordinates are defined as positive. Numerical labeling serves as identification for the atomic displacements shown in Table S1. Symmetry-paired atoms are denoted with a *.

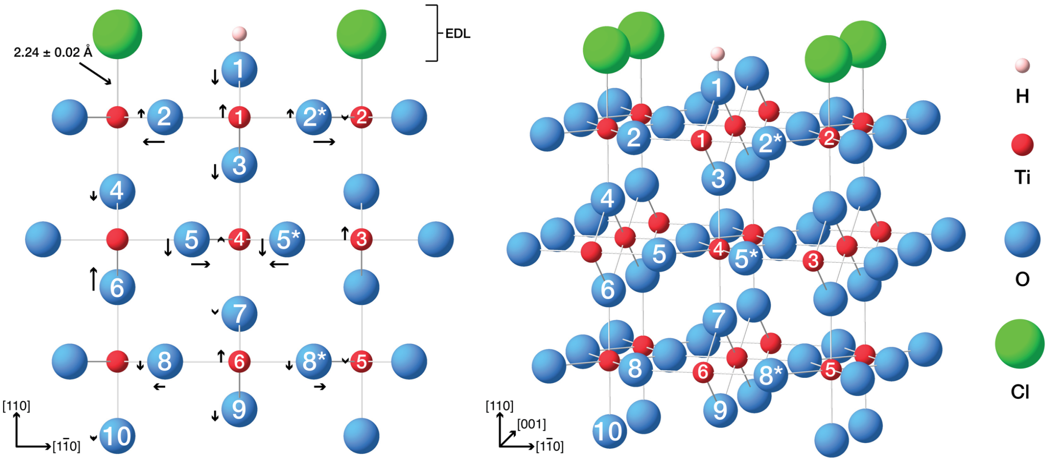

Figure S7: As S6 for the TiO_2(110)/ 0.1 M HCl interface. (a) Side view, and (b) tilted view of the interface structure. H atom positions are guided by theoretical calculations. The black arrows represent the relative magnitude and direction of atom displacements with respect to bulk lattice positions. The indicated azimuth defines the x, y, and z directions along which the atomic coordinates are defined as positive. Numerical labeling serves as identification for the atomic displacements shown in Table S1. Symmetry-paired atoms are denoted with a *.

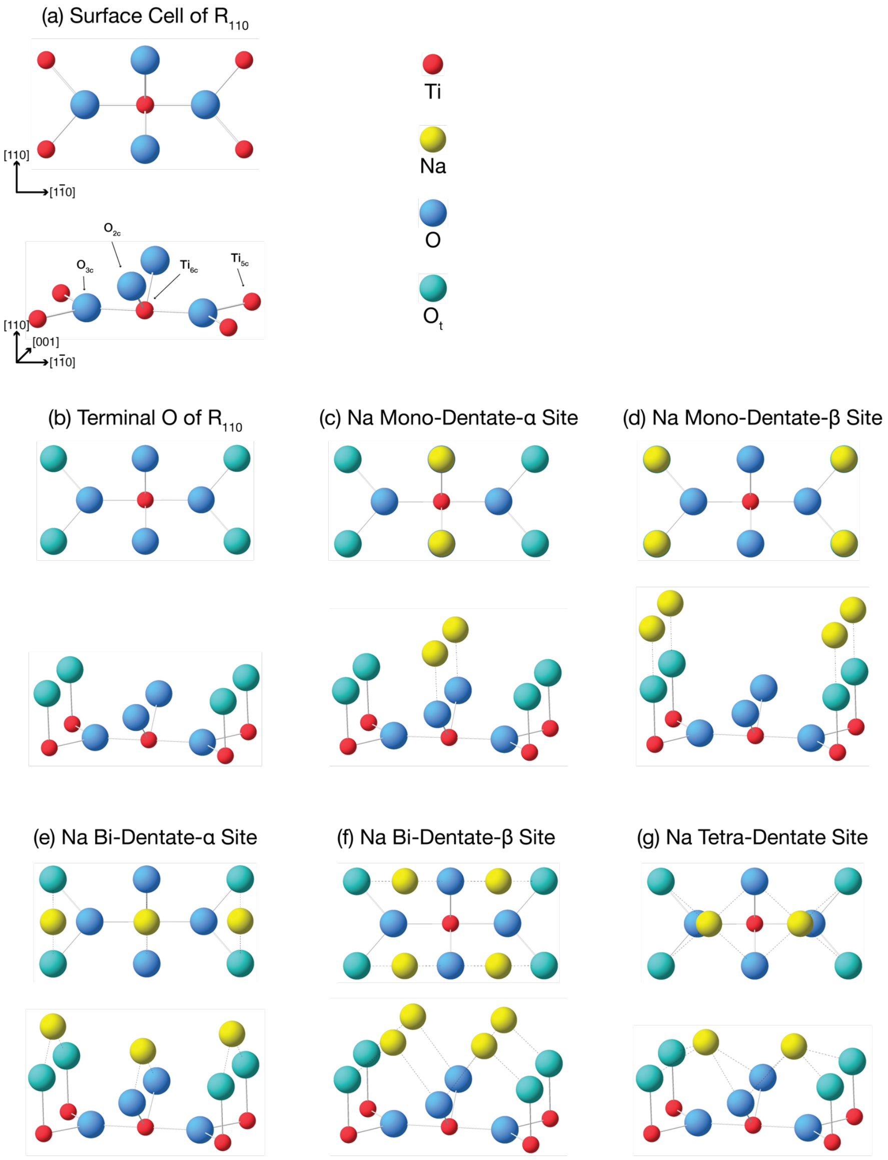

Figure S8: On-top and side view ball and stick models illustrating (a) the surface cell of TiO_2(110) (R_{110}), (b) with terminal OH (O_t) expected at pH 13, and (c-g) different high symmetry adsorption sites for Na on TiO_2(110).

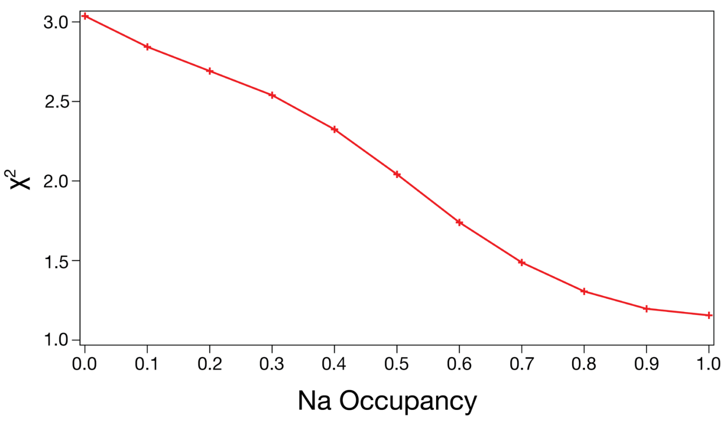

Figure S9: χ^2 variation Na occupancy (ML) for the $TiO_2(110)$ /0.1 M NaOH interface. All parameters were optimized after fixing the occupancy.

Atom Label	Displacements (Å)			
	TiO_2(110) / **UHV (Clean Surface)**		TiO_2(110) / **Electrolyte Interfaces**	
	Δ [110] [Nadeem : Cabailh[3] : Hussain[2]]	Δ [1$\bar{1}$0] [Nadeem : Cabailh[3] : Hussain[2]]	Δ [110] [$H_2O_{(l)}$[2]: 0.1 M NaOH : 0.1 M HCl]	Δ [1$\bar{1}$0] [$H_2O_{(l)}$[2]: 0.1 M NaOH : 0.1 M HCl]
O 1	0.10 ± 0.01 : 0.10 ±0.04 : 0.10 ± 0.04	-	0.08 ± 0.04 : 0.09 ± 0.01 : -0.08 ± 0.02	-
O 2*	0.13 ± 0.01 : 0.17 ±0.03 : 0.13 ± 0.03	0.03 ± 0.00 : 0.01 ± 0.05 : 0.02 ± 0.04	0.08 ± 0.03 : 0.13 ± 0.01 : 0.05 ± 0.01	0.01 ± 0.05 : 0.12 ± 0.01 : -0.13 ± 0.03
Ti 1	0.20 ± 0.01 : 0.25 ±0.01 : 0.17 ± 0.01	-	0.09 ± 0.01 : 0.08 ± 0.01 : 0.07 ± 0.01	-
Ti 2	-0.11 ± 0.01 : -0.11 ±0.01 : -0.06 ± 0.01	-	0.03 ± 0.01 : 0.08 ± 0.01 : -0.01 ± 0.01	-
O 3	0.08 ± 0.01 : 0.07 ±0.04 : 0.07 ± 0.04	-	0.07 ± 0.03 : 0.12 ± 0.01 : -0.08 ± 0.02	-
O 4	0.04 ± 0.01 : 0.00 ±0.03 : 0.02 ± 0.03	-	0.05 ± 0.05 : 0.04 ± 0.01 : -0.06 ± 0.02	-
O 5*	0.07 ± 0.01 : 0.04 ±0.03 : 0.03 ± 0.03	0.02 ± 0.00 : 0.05 ± 0.05 : 0.02 ± 0.03	0.06 ± 0.03 : 0.08 ± 0.01 : -0.10 ± 0.01	0.05 ± 0.05 : 0.05 ± 0.01 : 0.10 ± 0.02
Ti 3	-0.01 ± 0.01 : -0.08 ±0.01 : -0.02 ± 0.01	-	0.06 ± 0.01 :0.07 ± 0.01 : 0.07 ± 0.01	-
Ti 4	0.10 ± 0.01 : 0.19 ±0.01: 0.12 ± 0.01	-	0.04 ± 0.01 : 0.04 ± 0.01 : 0.02 ± 0.01	-
O 6	0.06 ± 0.01 : 0.01 ±0.04 : 0.04 ± 0.04	-	0.08 ± 0.05 : 0.01 ± 0.01 : 0.13 ± 0.02	-
O 7	0.01 ± 0.01 : 0.01 ±0.04 : 0.01 ± 0.04	-	0.06 ± 0.05 : 0.00 ± 0.01 : -0.01 ± 0.02	-
O 8*	0.03 ± 0.01 : 0.01 ±0.03 : 0.01 ± 0.05	-0.02 ± 0.00 : -0.03 ± 0.05 : -0.02 ± 0.05	0.05 ± 0.03 : 0.04 ± 0.01 : -0.05 ± 0.01	-0.03 ± 0.02 : -0.05 ± 0.01 : -0.06 ± 0.02
Ti 5	0.07 ± 0.01 : 0.08 ±0.01 : 0.05 ± 0.01	-	-0.04 ± 0.01 : 0.06 ± 0.01 : -0.01 ± 0.01	-
Ti 6	0.00 ± 0.01 : -0.04 ±0.01 : -0.02 ± 0.01	-	0.08 ± 0.01 : 0.06 ± 0.01 : 0.07 ± 0.01	-
O 9	0.05 ± 0.01 : 0.02 ±0.04 : 0.03 ± 0.03	-	0.04 ± 0.04 : 0.02 ± 0.01 : -0.06 ± 0.02	-
O 10	-0.01 ± 0.01 : -0.02 ±0.04 : -0.01 ± 0.03	-	0.03 ± 0.03 : -0.01 ± 0.01 : -0.02 ± 0.02	-

Table S1: Surface atomic displacements away from the bulk terminated structure of TiO_2(110) obtained via SXRD for an as-prepared surface and its interface with 0.1 M NaOH and 0.1 M HCl. Positive or negative displacements indicate those parallel or anti-parallel to the directions of the coordinate axis defined in Figures S6 and S7. The surface atomic displacements for the TiO_2(110) interfaces with 0.1 M NaOH and 0.1 M HCl are significantly modified from those of the as-prepared surfaces. In particular, the displacement of the Ti atoms in the first few layers is reduced. This phenomenon has been previously observed at the TiO_2(110)/$H_2O_{(l)}$ interface[2]. For the 0.1 NaOH interface, surface O atoms largely adopt similar positions to the as-prepared surface except for the O 2* atom in the Δ [1$\bar{1}$0] direction, the large displacement arising from its bonding to Na. For the 0.1 M HCl interface, surface O atoms are displaced towards the bulk, which will arise from the presence of Cl atoms on Ti_{5c}.

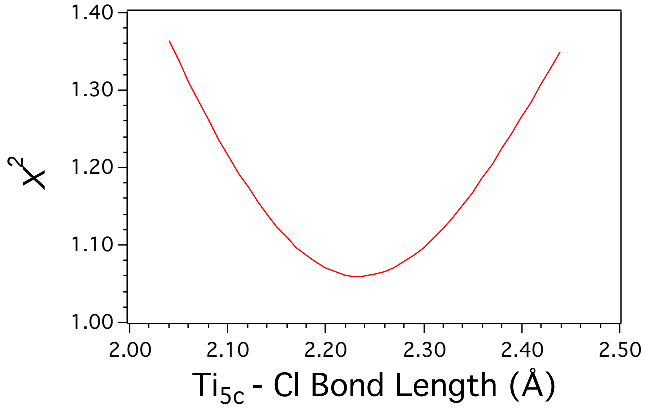

Figure S10: χ^2 variation with Ti_{5c}-Cl bond length for the $TiO_2(110)$ /0.1 M HCl interface.

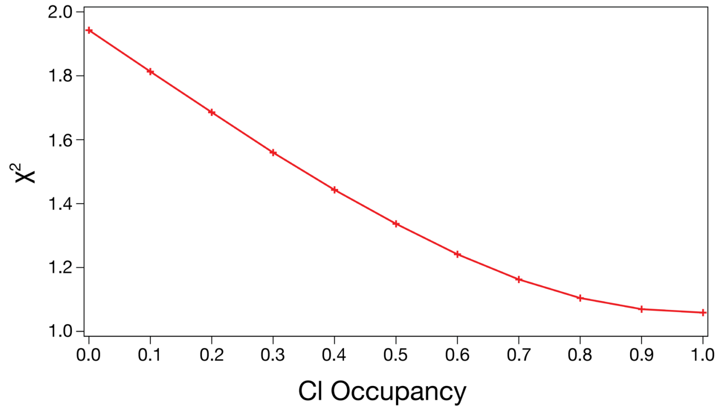

Figure S11: χ^2 variation with Cl occupancy (ML) for the $TiO_2(110)$ /0.1 M HCl interface. All parameters were optimized after fixing the occupancy.

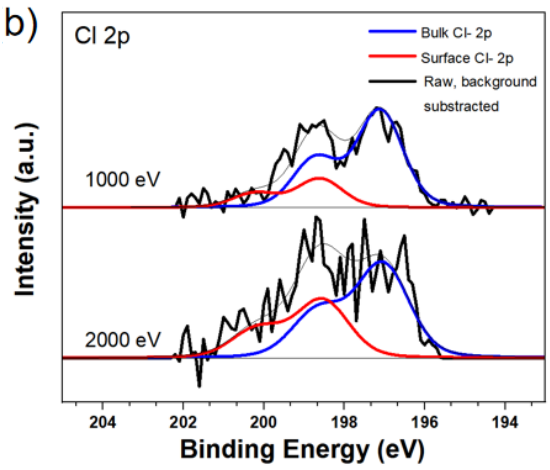

Figure S12: Ambient pressure XPS spectra were recorded from a 3.5 nm thick layer of 0.1 M HCl on TiO_2(110) using photon energies of 1000 eV and 2000 eV on beamline B07 at Diamond Light Source. Two Cl 2p spin-orbit split doublets are observed. The lower binding energy doublet is identified with Cl in the wetting layer and the higher binding energy doublet from Cl in the contact layer on TiO_2(110) using the photon energy dependence of electron escape depth[4].

Table S2: Calculated Bader charges for snapshots of the water/NaOH/TiO_2 and water/HCl/TiO_2 interfaces. Labels of the substrate atoms are shown in Figures S6 and S7.

Atom Label	Bader charges			
	NaOH	NaOH; Ti interstitials	HCl	HCl; Ti interstitials
Na/Cl	0.85	0.86	-0.68	-0.55
O_{water}	-1.23	-1.25	-1.23	-1.24
H_{water}	0.61	0.62	0.62	0.62
O_t	-1.24	-1.20		
HO_{br}			0.61	0.66
O 1	-1.31	-1.25	-1.07	-1.06
O 2*	-1.22	-1.21	-1.17	-1.17
Ti 1	2.31	2.31	2.32	2.32
Ti 2	2.31	2.28	2.30	2.28
O 3	-1.16	-1.17	-1.17	-1.16
O 4	-1.17	-1.15	-1.16	-1.16
O 5*	-1.17	-1.19	-1.17	-1.19
Ti 3	2.32	2.31	2.34	2.32
Ti 4	2.34	2.33	2.33	2.31
O 6	-1.17	-1.21	-1.16	-1.20
O 7	-1.16	-1.19	-1.16	-1.18
O 8*	-1.17	-1.19	-1.17	-1.19
Ti 5	2.34	2.25	2.33	2.25
Ti 6	2.33	2.33	2.33	2.32
O 9	-1.13	-1.15	-1.14	-1.15
O 10	-1.18	-1.19	-1.18	-1.19

Table S3: Bond Length Comparison (DFT vs SXRD)

Bond Length (Figure 2(b))	SXRD (Å) ±0.01Å	DFT
Ti_{5c} - O_t	2.03	1.82±0.05
O_t - Na	2.40	2.30±0.11
O_{br} - Na	2.77	2.59±0.17
H_2O - Na	2.80	2.78±0.42
Ti - Cl	2.24	2.37±0.15

Mov 1: Ab initio molecular dynamics simulation of the TiO_2(110)/NaOH interface. The video shows a side view of the 35 ps long trajectory, sampled every 50 fs. Ti interstitials can be seen between the second and third surface layers. The black lines indicate unit cell boundaries. Ti: red, O: blue, O_t: light green, O_{water}: dark green, H: white, Na: yellow.

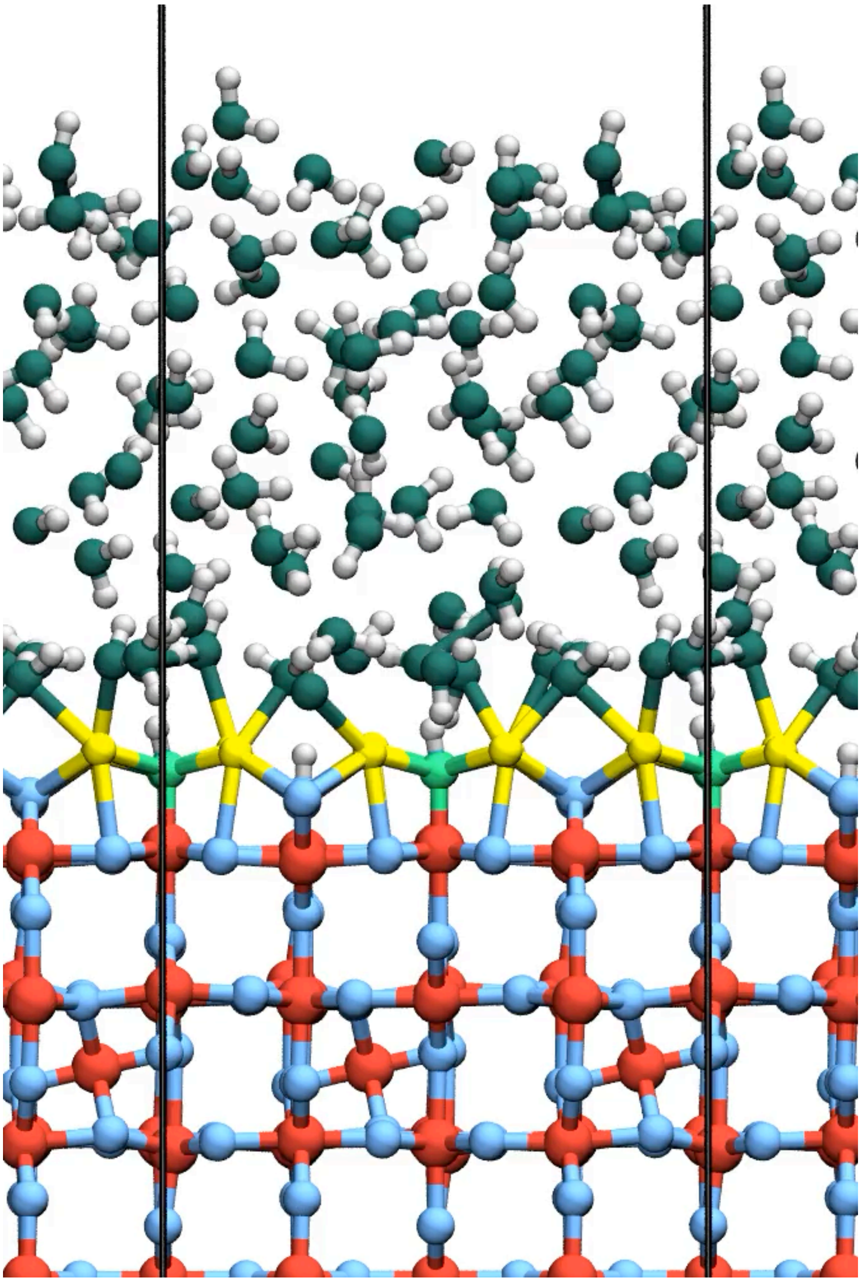

Mov 2: Ab initio molecular dynamics simulation of the TiO_2(110)/HCl interface. The video shows a side view of the 33 ps long trajectory, sampled every 50 fs. Ti interstitials can be seen between the second and third surface layers. Cl ions remain at the interface, while protons are transferred between O_{br} and O_{water}. The black lines indicate unit cell boundaries. Ti: red, O: blue, O_{water}: dark green, white, Cl: green.

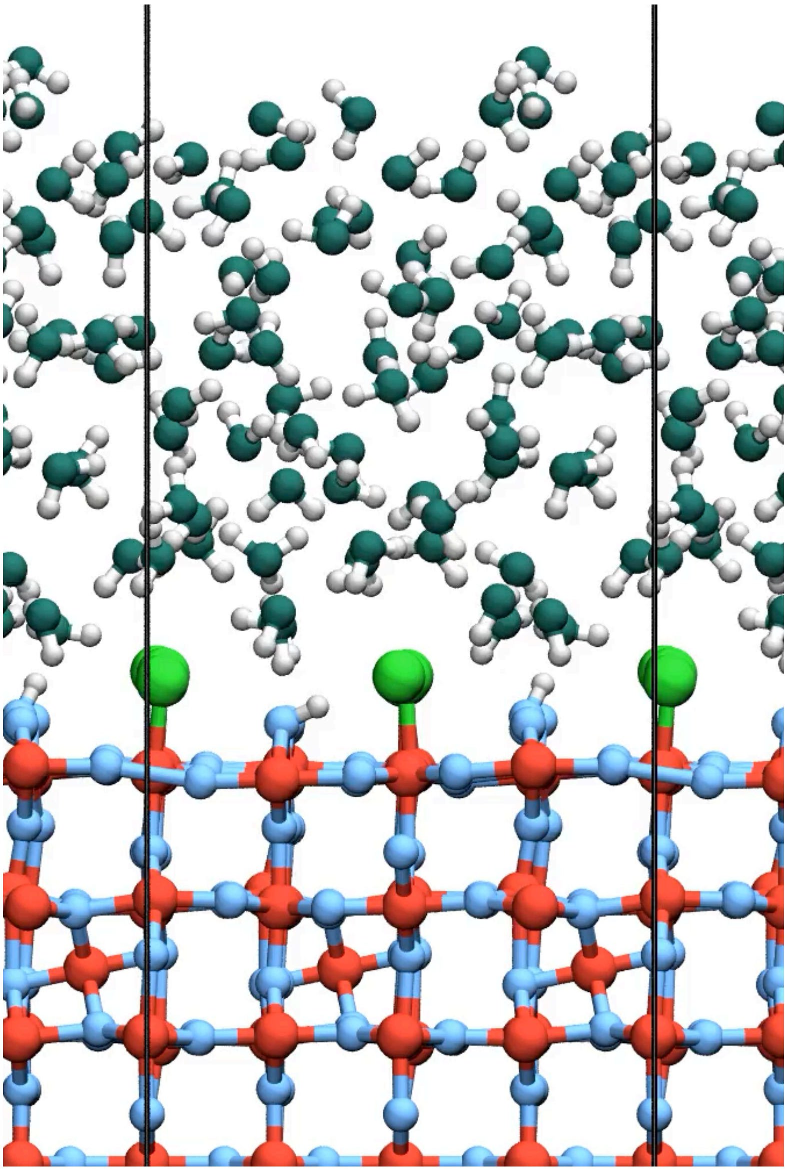

References

1. Treacy, J. Synchrotron studies of TiO_2 single crystal surfaces. **2014**, PhD thesis, The University of Manchester. Available at: https://eur01.safelinks.protection.outlook.com/?url=https%3A%2F%2Furldefense.com%2Fv3%2F__https%3A%2F%2Fresearch.manchester.ac.uk%2Fen%2FstudentTheses%2Fsynchrotron-studies-of-tio2-single-crystal-surfaces__%3B!!D9dNQwwGXtA!XC0sXnmghLgh6rMlZdJJMkKfoMAcqKisCk4ozRCP7C94QdR3l-mjVNoE37-0cxxNKtrUW6d_Mfa0iaM7Us2eGg-iPkeQFI_icQ%24&data=05%7C02%7Cg.thornton%40ucl.ac.uk%7C2f4de7ec06d44c9a040508dcf8f605a9%7C1faf88fea9984c5b93c9210a11d9a5c2%7C0%7C0%7C638658982768982121%7CUnknown%7CTWFpbGZsb3d8eyJWIjoiMC4wLjAwMDAiLCJQIjoiV2luMzIiLCJBTiI6Ik1haWwiLCJXVCI6Mn0%3D%7C0%7C%7C%7C&sdata=2bNItaK2xW6sX7TDu%2BPkYbkvSK%2FbJ%2BeJcgOet6%2BgAAw%3D&reserved=0
2. Hussain, H.; Tocci, G; Woolcot, T; Torrelles, X.; Pang, C.L.; Humphrey, D.S.; Yim, C.M.; Grinter, D.C.; Cabailh, G.; Bikondoa, O.; Lindsay, R.; Zegenhagen, J.; Michaelides, A.; Thornton, G. Structure of a model TiO_2 photocatalytic interface. *Nat. Mater.* **2017**, 461-466.
3. Cabailh, G.; Torrelles, X.; Lindsay, R.; Bikondoa, O.; Joumard, I.; Zegenhagen, J.; Thornton, G. Geometric structure of TiO_2(110)(1×1): achieving experimental consensus. *Phys. Rev. B* **2007**, 241403.
4. Yu, J., Byrne, C., Imran, J., Henderson, Z., Holt, K.B., Large, A.I., Held, G., Walton, A., Thornton, G. Operando characterization of electrochemistry at the rutile TiO_2(110)/0.1 M HCl interface using ambient pressure XPS. *J. Phys. Chem. C*, in press.
